# A Tensorized Multitask Deep Learning Network for Progression Prediction of Alzheimer’s Disease

**DOI:** 10.3389/fnagi.2022.810873

**Published:** 2022-05-06

**Authors:** Solale Tabarestani, Mohammad Eslami, Mercedes Cabrerizo, Rosie E. Curiel, Armando Barreto, Naphtali Rishe, David Vaillancourt, Steven T. DeKosky, David A. Loewenstein, Ranjan Duara, Malek Adjouadi

**Affiliations:** ^1^Center for Advanced Technology and Education, Florida International University, Miami, FL, United States; ^2^Harvard Ophthalmology AI Lab and Harvard Medical School, Schepens Eye Research Institute, Massachusetts Eye and Ear, Boston, MA, United States; ^3^Center for Cognitive Neuroscience and Aging, Psychiatry and Behavioral Sciences, University of Miami School of Medicine, Miami, FL, United States; ^4^Florida Alzheimer’s Disease Research Center, University of Florida, Gainesville, FL, United States; ^5^Department of Neurology, University of Florida, Gainesville, FL, United States; ^6^Department of Applied Physiology and Kinesiology, University of Florida, Gainesville, FL, United States; ^7^Wien Center for Alzheimer’s Disease and Memory Disorders, Mount Sinai Medical Center, Miami Beach, FL, United States

**Keywords:** Alzheimer’s disease, multitask learning, prediction, longitudinal regression, progression, neural network

## Abstract

With the advances in machine learning for the diagnosis of Alzheimer’s disease (AD), most studies have focused on either identifying the subject’s status through classification algorithms or on predicting their cognitive scores through regression methods, neglecting the potential association between these two tasks. Motivated by the need to enhance the prospects for early diagnosis along with the ability to predict future disease states, this study proposes a deep neural network based on modality fusion, kernelization, and tensorization that perform multiclass classification and longitudinal regression simultaneously within a unified multitask framework. This relationship between multiclass classification and longitudinal regression is found to boost the efficacy of the final model in dealing with both tasks. Different multimodality scenarios are investigated, and complementary aspects of the multimodal features are exploited to simultaneously delineate the subject’s label and predict related cognitive scores at future timepoints using baseline data. The main intent in this multitask framework is to consolidate the highest accuracy possible in terms of precision, sensitivity, F1 score, and area under the curve (AUC) in the multiclass classification task while maintaining the highest similarity in the MMSE score as measured through the correlation coefficient and the RMSE for all time points under the prediction task, with both tasks, run simultaneously under the same set of hyperparameters. The overall accuracy for multiclass classification of the proposed KTMnet method is 66.85 ± 3.77. The prediction results show an average RMSE of 2.32 ± 0.52 and a correlation of 0.71 ± 5.98 for predicting MMSE throughout the time points. These results are compared to state-of-the-art techniques reported in the literature. A discovery from the multitasking of this consolidated machine learning framework is that a set of hyperparameters that optimize the prediction results may not necessarily be the same as those that would optimize the multiclass classification. In other words, there is a breakpoint beyond which enhancing further the results of one process could lead to the downgrading in accuracy for the other.

## Introduction

Extensive research has focused lately on using different machine learning techniques for the diagnosis and prognosis of AD. However, a retrospective of previous studies on multimodal datasets reveals some inconsistencies in modeling the relationship between the many features captured from the different recording modalities. Although several linear methods have been previously reported in the literature with the ability to linearly fuse the information from different modalities (Perrin et al., 2009), several authors have also suggested different non-linear approaches to fuse the multimodal biomarkers (Wang et al., 2012, 2018c; Huang et al., 2016; Tong et al., 2017).

The relatively low accuracy of the classification and regression techniques in delineating converter from non-converter groups and Mild Cognitive Impairment (MCI) from Cognitively Normal (CN) draws our attention to the diversity and heterogeneity of the potential features that could be extracted from the multimodal and multiclass AD datasets (Pellegrini et al., 2018). For example, Wei et al. (2016) proposed a classification method to distinguish non-converter MCI (MCI-NC) from converter MCI (MCI-C) by using an SVM classifier over features that are a combination of FreeSurfer-derived MRI features and nodal features derived from the thickness network. In another recent study (Lin et al., 2020), the authors developed an extreme learning machine (ELM grading method to efficiently fuse multimodal data and predict MCI-to-AD conversion within a 3-year duration. In Huang et al. (2021), subjects are classified as healthy controls, subjective cognitive decline (SCD), or amnestic mild cognitive impairment (aMCI) based on SVM and features extracted from white matter. The ability to detect subtle changes that could lead to a more accurate classification of MCI stable from MCI converter remains extremely challenging. This is why most machine learning models opt for binary classification as an initial step for determining relevant indicators of the model how to best separate these two very difficult MCI subgroups (Tolonen et al., 2018; Gupta et al., 2020).

With the advent of deep learning and their multilayer structure at elucidating lingering abstract steps of machine learning, especially as it pertains to the extraction of relevant features in multimodal multiclass classification and regression processes, there is great interest in their application to brain research in general and complex neurodegenerative diseases like Alzheimer’s disease (Liu et al., 2016; Sarraf and Tofighi, 2016; Zhang et al., 2017; Amoroso et al., 2018; Choi and Jin, 2018; Fisher et al., 2018; Lu et al., 2018; Wang et al., 2018a). In Jo et al. (2019), an extensive review for applying deep learning in neuroimaging data is provided, with a focus placed on the diagnosis and prognosis of AD and its prodromal stages. In Kang et al. (2020), a CNN-based classifier with a specific regularization technique is proposed to distinguish early MCI vs. CN subjects using structural MRI and diffusion tensor imaging (DTI) as input to their CNN-based model. Liu et al. (2018b) proposed a cascaded CNN that makes use of multimodal patch-based features from different regions of the brain. Using MRI and PET images, their deep 3D-CNN algorithm could achieve good binary accuracy in differentiating AD vs. CN, progressive MCI vs. CN, and stable MCI vs. CN.

Autoencoders have also been explored for their ability to extract high-level complex patterns embedded in the features to enhance classification accuracy (Suk and Shen, 2013; Liu et al., 2016). For example, in Jha and Kwon (2017), a sparse autoencoder is used for binary classification of AD from cognitively normal (CN) subjects. The use of Recurrent Neural Networks has been proposed by Wang et al. (2018b) to predict a future stage of the patient using historical clinical records. A related study (Liu et al., 2018b) proposed a combination of CNN and Recurrent Neural Network (RNN) for feature extraction and classification. Considering the large size of PET images, instead of using 3D CNN, they employed 2D CNN to extract features from 2D PET slices. The extracted features were then used through gated recurrent units (GRU) for the classification of AD and MCI subjects from the CN group.

With significant efforts made for predicting cognitive scores to track disease progression and for anticipating a diagnosis label at future timepoints to determine a future stage of the disease, the correlation between categorical and numerical variables brings the potentially open question of whether jointly learning based approaches could leverage the learning performance of both classification and regression tasks. Liu et al. (2018c) proposed the use of a CNN model for joint regression and classification tasks. Using their deep multitask multichannel learning (DM^2^L) framework, they reached an accuracy of 51.8% in a four-class classification process. In another study by Zhu et al. (2016a), multimodal feature fusion has been explored through a sparse multitask learning process to predict ADAS-Cog, MMSE, and AD stages simultaneously. Another attempt by Shi et al. (2018) is made to perform both tasks of binary and multiclass classification, where a two-stage stacked deep polynomial network is used, obtaining an accuracy of 55.34% in multiclass classification with higher accuracies obtained for binary classification. The multitarget regression approach can also be categorized in this domain of application. In Zhen et al. (2018), the authors encoded the inter-target correlation and the relationship between the input and output space *via* low-rank learning. In a study by Zhang and Shen (2013), a multi-modal multi-task (M3T) learning framework is used for the prediction of multiple clinical variables of MMSE and ADAS-cog from a multimodal dataset. With similar objectives (Zhu et al., 2014), utilized a matrix-similarity-based loss function combined with group lasso to select the best features for both classification and regression tasks.

In this study, a novel neural network architecture, structured as a Kernelized and Tensorized Multitask network (KTMnet) is proposed for processing two joint tasks of classification and longitudinal prediction simultaneously. This network uses dense layers to first extract features from each modality separately, then uses Gaussian kernel layers and tensorization over the modality fused feature space to non-linearly map the data from a low-dimensional space to a high-dimensional space. Empirical results show enhanced performance in comparison to all related methods reviewed in this article, especially when delineating the challenging group of MCI (converters and non-converters) from CN in a multiclass classification scenario.

## Materials and Methods

### Subjects

The clinical data used in the preparation of this article were obtained from the Alzheimer’s Disease Neuroimaging Initiative (ADNI) database (adni.loni.usc.edu). A total number of 1,117 individuals consisting of 632 males and 485 females were considered for this study. The average age is 73.84 with total average years of education of 16.04. The average MMSE score of the population is 27.44 at baseline and 27.06, 26.82, and 26.02 at the next 6 and 12, and 24 months, respectively. At each follow-up visit, participants were labeled as AD, MCI (Mild Cognitive Impairment), and CN, and those participants from the MCI stage that converted to AD are labeled as the MCI to the AD group. The demographics of the subjects are given in Table 1.

**TABLE 1:** Demographic characteristics of subjects used in this study.

Parameter	Value	Total	Alzheimer	MCI-C	MCI-NC	Control
Subjects	Number	1,117	157	191	441	328
Gender	f/m	485/632	73/84	75/116	184/257	153/175
Age	Year (mean ± std)	73.84 ± 7.07	76.77 ± 6.99	73.86 ± 7.47	70.85 ± 7.19	75.01 ± 5.71
Education	Year (mean ± std)	16.04 ± 2.78	14.63 ± 3.15	16.09 ± 2.74	16.09 ± 2.63	16.36 ± 2.68
MMSE	Number (mean ± std)	27.43 ± 2.46	23.24 ± 1.96	27.23 ± 1.75	28.30 ± 1.59	29.15 ± 1.01
CDR	Number (mean ± std)	1.25 ± 1.36	3.98 ± 1.51	1.62 ± 0.92	1.24 ± 0.74	0.03 ± 0.13

*Label f/m stands for the number of females in comparison to males. Age, years of education, MMSE, and CDR of subjects in each category are presented by mean ± standard variation of that variable.*

### Problem Description

In longitudinal AD studies, disease progression can be gauged *via* screening the categorical or numerical labels of participants through time. The categorical labels in ADNI are AD, MCI (including the converter and non-converter groups), and CN. On the other hand, there are also numerical measurements needed to assess cognitive impairment, which augment the in-depth analysis of the data. Mini-Mental State Examination or MMSE is the best-known clinical AD predictor that is accepted and used worldwide. While predicting the diagnosis labels is accomplished through classification methods and predicting the numerical value of neuropsychological test scores is performed through regression models, the underlying features for both tasks are constructed from similar sets of measurements. This relationship between these two types of modeling methods motivated researchers to train these highly interrelated tasks of regression and classification through multitask learning.

**FIGURE 1:**
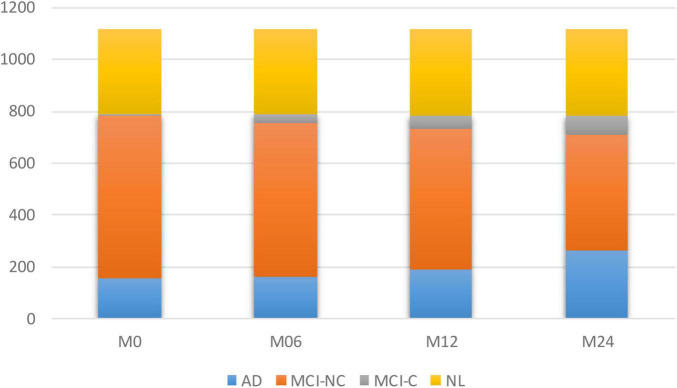
Number of subjects in each of the four subgroups of AD at different time points.

To model the progression of AD, a time frame of 24 months has been considered to assess the conversion prospects of the MCI group into AD. Therefore, only those subjects that completed a baseline scan (M0) and showed up for a follow-up visit 6 months later (M6), 12 months later (M12), and 24 months later (M24) were considered. Studying longitudinal AD cohorts could improve our understanding of AD pathogenesis. While most patients that have been diagnosed as belonging to the intermediate stage of MCI have been known to progress toward the AD stage, there is some evidence that some of them might stabilize at the MCI stage. However, the different conversion slopes for the different individuals suggest that this stable group is converting into AD in a much longer time frame. Figure 1 shows the number of subjects in each category of AD over the 24-month duration.

**FIGURE 2:**
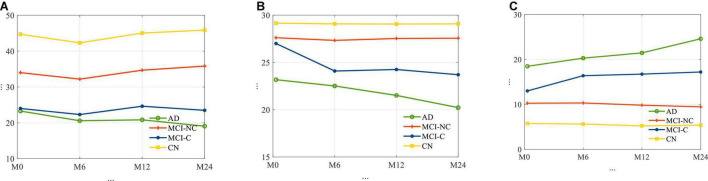
The average trajectories of **(A)** RAVLT, **(B)** MMSE, and **(C)** ADAS11 score for subjects for four different classes of AD.

The average longitudinal changes of neuropsychological test scores for the 4 subgroups are shown in Figure 2. It is observed that for AD and MCI-C populations, the mean of the cognitive test score for these groups has decreased over time by 13 and 12.7%, respectively. This suggests a continuous decline in health status and thus the need for predicting cognitive decline as early as possible.

### Problem Formulation

**FIGURE 3:**
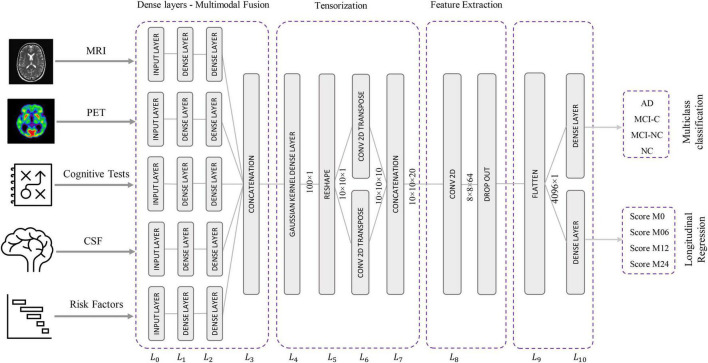
Design architecture of the proposed KTM network.

The proposed Kernelized and Tensorized Multitask network (KTMnet) shown in Figure 3 is structured to estimate the progression of Alzheimer’s disease by predicting the categorical and numerical labels simultaneously. Let *y*_*r*_ be the sets of longitudinal neuropsychological test scores (MMSE) for the regression task (Task 1) and *y*_*c*_ be the sets of categorical labels for the classification task (Task 2). The input space for both tasks is the multimodal features of {*x*_m_1__, *x*_*m*_2_,_ *x*_*m*_3_,_ *x*_*m*_4_,_ *x*_*m*_5__}, in which the vector *x*_*m*_*i*__ comprises the extracted measurements from modality *i*. Note that these input features are extracted from MRI, PET, CSF, cognitive tasks, and the risk factors at baseline. Hence, vectors *y*_*r*_ and *y*_*c*_ for this study can be established as *y*_*r*_ = [*Score*_*M*0_, *Score*_*M*6_, *Score*_*M*12_, *Score*_*M*24_]′ and *y*_*c*_ = [*AD*, *MCI* − *C*, *MCI* − *NC*, *CN*]′, where *MCI-C* and *MCI-NC* define the MCI converter and non-converter groups, with the prime symbol (’) defining the transpose function. The risk factor parameters considered are age, years of education, sex, and APOE4. The overall objective function, in this case, could be modeled as an algorithm in which *y*_*r*_ = *E*_*r*_(*x*_*m*_1__, x_*m*_2__, x_*m*_3__, x_*m*_4__, *x*_*m*_5__) and *y*_*c*_ = *E*_*c*_(*x*_*m*_1_,_ x_*m*_2__, x_*m*_3__, x_*m*_4__, *x*_*m*_5__) with *E*_*r*_ and *E*_*c*_ being the corresponding estimators. The architecture of the proposed KTMnet method is shown in Figure 3.

The proposed network consists of a series of operations defined through Eqs. (1, 2). Feature representation, modality fusion, and tensorization have been incorporated in an end-to-end artificial neural network to harness the advantage of performing regression and classification tasks jointly in a unified framework. The multitask framework aims to make use of the features extracted from each modality through modality fusion and tensorization to secure optimal accuracy for both prediction and multiclass classification when such tasks are run simultaneously. First, the feature vectors of each modality would be extracted by *F*_*m_i_*_ and then all the features from different modalities will be fused by function *f*. Next, a 3D tensorization (T) is applied to the fused feature vector to represent higher-order relations between features. Finally, tensor features will be extracted by *F* and fed to the regressor function *f*_*r*_ and classifier function *f*_*c*_.


(1) $$Task1:{\hat{y}}_{r}=f_{r}(F(\text{T}(f(F_{m_{1}}\left(x_{m_{1}}\right),F_{m_{2}}\left(x_{m_{2}}\right),F_{m_{3}}\left(x_{m_{3}}\right),F_{m_{4}}\left(x_{m_{4}}\right),F_{m_{5}}\left(x_{m_{5}}\right))))$$

(2) $$Task2:{\hat{y}}_{c}=f_{c}(F(\text{T}(f(F_{m_{1}}\left(x_{m_{1}}\right),F_{m_{2}}\left(x_{m_{2}}\right),F_{m_{3}}\left(x_{m_{3}}\right),F_{m_{4}}\left(x_{m_{4}}\right),F_{m_{5}}\left(x_{m_{5}}\right))))$$

The loss function used to calibrate jointly the longitudinal regression and classification tasks is as follows:


(3) $$L\,o\,s\,s=\alpha \times M\,S\,E\,\;\left(y_{r},{\hat{y}}_{r}\right)+\,\beta \times \,l\,\left(y_{c},\,{\hat{y}}_{c}\right)$$

in which *y* is the target value and $\hat{y}$ is the value predicted by the network. The MSE is the mean square error for the regression task defined as:


(4) $$M\,S\,E=\frac{1}{N}\,\sum_{i=1}^{N}{\left(y_{r\,i}-\,{\hat{y}}_{r\,i}\right)}^{2}$$

And the categorical cross-entropy of $l\,\left(y_{c},\,{\hat{y}}_{c}\right)$ is defined as:


(5) $$l\,\left(y_{c},\,{\hat{y}}_{c}\right)=-\,\frac{1}{N}\,\,\sum_{i=1}^{N}\left[y_{c\,i}\,\log\hat{y_{c\,i}}+\left(1-\,y_{c}\right)\,\log\left(1-\hat{y_{c}}\right)\right]$$

where N is the number of observations and c is the number of categories assigned to the class label.

### Network Architecture

This network architecture relies on convolutional neural layers to jointly perform the processes of tensorization and feature extraction. Given the schematic diagram of the network shown earlier in Figure 3, the main properties of the proposed network are as described in the following subsections.

#### Modality Fusion

The relational correlation of features within each modality and between the different modalities remains an important subject in developing robust prediction algorithms. The importance of using and fusing relevant information from different modalities to improve classification is well documented in the literature, and some studies have shown significant improvement in comparison to relying on a single modality. For this reason, modality fusion has also been considered in the proposed network to incorporate the advantages of intra-modality and inter-modality feature representation. First, the network starts by transforming the raw features into a primary single modality representation space using fully connected layers. Two fully connected layers of L0 and L1 are then used to transform the extracted features from MRI, PET, CSF, neurocognitive measurements, and risk factor parameters into an initial intra-modality feature-space representation. Let n_mod be the length of the input feature vector of named modality mod, then L0 is the input layer for each modality with n_mod nodes. These single modality features are then processed *via* two fully connected layers of L1 and L2 with [2 × n]_mod and n_mod nodes followed by linear activation function layers. The intermodality feature space is then initiated by integrating the previous fully connected layers in L3, which concatenates the outputs of the L2 layer to create the new feature vector.

#### Tensorization

Complementary and shared information found in features from different modalities is an essential part of reliably modeling the progression of neurodegenerative diseases. However, concatenating the features from different modalities and processing them using a simple network will not consider the inhomogeneity of the multimodal dataset. Therefore, it is reasonable to transform the feature space into a higher dimensional receptive field to enable the network to find more meaningful relationships. A non-linear mapping function can map linearly inseparable data from a low-dimensional space into a high-dimensional space where it becomes possible to linearly separate the mapped data. The Gaussian kernel function is a representative function that is commonly used and is also adapted in neural networks (Srisuphab and Mitrpanont, 2009; Fei et al., 2016).

Tensorization is thus defined as transforming or mapping the lower-order data to higher-order data to improve the process of generalization afforded at this higher-order (Debals and De Lathauwer, 2015; Novikov et al., 2015). This means that when the data is not providing a satisfactory feature representation in a lower-dimensional space, transferring it to a higher dimensional space may improve the data analysis with the potential for retrieving hidden information in that same data. As an example, a vector can thus be reshaped into a 2D matrix or a 3D tensor of any arbitrary shape with width, height, and depth of W × H × D dimensions. Similarly, a matrix can also be reshaped into a higher-order tensor, by reshaping each column to a tensor of order K and stacking the results along the K + 1 dimension.

In this new architecture, a dense layer with a Gaussian kernel is used for kernelization and a convolutional neural network is used for tensorization, and both are used to extract higher-order features from fused multimodal features. In this way, a tensor with the size of 10 × 10 × 20 is generated using the following procedure:

– L4 uses Gaussian dense layer to assist tensorization.
– L5 reshapes the 100-node output vector of layer L4 to create a 2D 10 × 10 tensor.
– L6 performs 2D transpose convolutional filtering with a kernel size of 3 × 3, a stride of 1, padding type of “same,” and linear activation function along with:
– 10 kernels with a dilation rate of 1.
– 10 kernels with a dilation rate of 2.
– Concatenation of the outputs from the two above dilation layers.

#### Feature Extraction

In this step, predictive features are extracted from the generated tensor. Since the feature extraction part is also based on 2D convolutional filtering with the network being trained in an end-to-end fashion, there is not a strong distinction for separating the network into the tensorization part and feature extraction part. The extracted features at the end of this stage make up the tensor. For this reason, 2D convolutional filtering is performed in L8 by using 64 filters with a kernel size of 4 × 4 and applying the ReLU activation function. A dropout rate of 10% is implemented to randomly deactivate the connection between the neurons during the training phase to overcome any potential for overfitting.

### Classification and Longitudinal Regression

This last component of the network is dedicated to classification and regression. For this reason, L9 flattens the output of the L8 layer to build a vector with the size 4,096 × 1. The output of the L12 layer is connected *via* two fully connected networks with an L1 regularizer to the two output layers (i.e., *y*_*r*_ and *y*_*c*_) in L10. Four nodes are assigned for the regression part, which has a ReLU activation function, and four nodes are assigned for the classification part with a Softmax activation function.

### Optimizer Selection

In deep learning, choosing the right optimization method is key to tuning an accurate model. During the training, weights are iteratively updated until the network converges to a minimum cost function. Small learning rates will keep updating the weights with smaller steps, which could consequently lead to a minimal loss function. Updating the weights by taking large scales comes with the risk of skipping over the optimal weights. Still, some measure of caution should be taken when assuming smaller steps, as there is a risk of being trapped into some local minima.

For the proposed network, after testing several common optimization methods for training, the adaptive Adam algorithm has been selected as the optimization method. Adam, developed by Kingma and Ba (2014), is one of the most common and adaptive optimizers used in deep learning applications, which adaptively approximates lower-order moments to yield an efficient and easy-to-tune solution. The adaptive learning rate is estimated by retaining an exponentially decaying average of previously squared gradients along with keeping the exponentially decaying averages of past gradients. Using this optimization approach with a learning rate of 0.001 and with exponential decay rates for the moment estimates β_*1*_ and β_2_ of 0.9 and 0.999, respectively, resulted in a robust trained network that consolidates high precision, sensitivity, F1 score, and area under the curve (AUC) in the multiclass classification task with high similarity in predicted vs. actual MMSE scores at all-time points in the prediction task.

Regularization and dropouts were used to minimize the likelihood of overfitting in layers L4, L8, and L9. Feature dimensionality reduction is exploited to implicitly select and extract features between L1 and L2 and between L9 and L10. While all network layers from L1 to L10 are extracting features, the main part of the tensorization process is assumed to take place in layers L5 through L8 based on transposed and dilated convolutional filtering.

In summary, the proposed structure of the network accomplishes both classification and longitudinal regression tasks by enabling the network to utilize the complementary/shared information in the extracted features space. Integrating these two challenging tasks within a unified framework elevated the accuracy and robustness of the model by taking into consideration the inter-relatability between tasks in a multitask process. For training the network, an end-to-end learning process has been used to learn from both feature representation and modality fusion simultaneously to address both regression and classification tasks.

## Preprocessing and Experimental Setup

### Preprocessing

The procedure for predicting disease progression requires considering additional constraints. Subsequently, only the subjects that have a baseline scan and who showed up for a follow-up visit at 6, 12, and 24 months later, were considered in this longitudinal data collection.

The following preprocessing steps are performed in this analysis:

– Exclude all subjects whose cognitive score or diagnosis label has not been reported.
– Exclude the Aβ, P-tau, or Tau values, reported out of range (e.g., > 1,300 or < 80 for Tau).
– Remove the predictive biomarkers of ADAS13, MoCA, and CDR, which are found to be highly correlated with the status or label of the subjects. This was done so as not to bias favorably our longitudinal regression results which involve predicting future MMSE scores.
– Perform mean centering and normalization of training and test data using mean and variance of training data (z-score).

At the end of these preprocessing steps, a total number of 1,117 subjects, among them 328 CN, 191 MCI-C, 441 MCI-CN, and 157 AD subjects were considered for this study. Table 2 provides an overview of the multimodal features used in this study.

**TABLE 2:** Summary of multimodal features used for training and testing the KTMnet dataset.

Source	Features
	
MRI	Ventricular volume, Hippocampus volume, Whole Brain volume, Entorhinal Cortical thickness, Fusiform, Middle temporal gyrus, and intracranial volume (ICV)
PET	FDG, Pittsburgh Compound-B (PIB), AV45
Cognitive test	Rey Auditory Verbal Learning Test (RAVLT Immediate, RAVLT Learning, RAVLT Forgetting, RAVLT Perc Forgetting), Functional Activities Questionnaires (FAQ), Everyday Cognition (Ecog) scales: (EcogPtMem, EcogPtLang, EcogPtVisspat, EcogPtPlan, EcogPtOrgan, EcogPtDivatt, EcogPtTotal, EcogSPMem, EcogSPLang, EcogSPVisspat, EcogSPPlan, EcogSPOrgan, EcogSPDivatt, and EcogSPTotal)
CSF	Amyloid Beta (ABETA), Phosphorylated Tau Protein (PTAU), and Total Tau Protein (TAU)
Risk factors	Age, gender, years of education, and APOE4

### Experimental Setup

Empirical evaluations were conducted on the Intel Xeon E7 with NVIDIA QUADRO M6000 GPU. The proposed network is implemented in Python with the Keras library (Chollet et al., 2015) using the TensorFlow backend (Abadi et al., 2016). For hyperparameter selection, a split of 15% of the data has been dedicated to threefold cross-validation trials, where the set of hyperparameters (including α and β in equation 3) that achieved the minimum bias and variance has been selected. The hyperparameters are the number of kernels used in L9, L10, L11 in the range of {256, 128, 64, 32, 16, and 8} and β in the range of {10, 20, and 200} in which grid search has been performed. After hyperparameter selection, similar to the approach utilized in Suk and Shen (2013), Liu et al. (2018b), and Cao et al. (2018), 10-fold cross-validation trials were performed on the remaining 85% of data to avoid the occurrence of bias within a lucky partitioning. In each round of training set, 10% of data has been utilized as a validation set for monitoring the training process to prevent the network from overfitting. A batch size of 150 and the maximum number of epochs of 200 were set for this process and the training is stopped by monitoring the loss of validation with 30 patience epochs. We performed two sets of experiments to analyze the contribution of this work for each of the prediction tasks for evaluation purposes.

#### Task 1: Regression Task for Prediction of Disease Progression

In the following experiments, the first task of our KTMnet model is the longitudinal prediction of trajectories of the MMSE score. The neuroimaging modalities of MRI and PET, the cerebrospinal fluid (CSF) biomarkers, genetic information, and cognitive assessment tests have been used to create the multimodal data. Since the state-of-the-art algorithms used different performance metrics, to benchmark our method with other methods, network performance is measured by the following common metrics:

The Root Mean Square Error is defined as follows:


(6) $$R\,M\,S\,E=\sqrt{\frac{1}{N}\,\sum_{i=1}^{N}{\left(Y_{i}-\,{\hat{Y}}_{i}\right)}^{2}}$$

The R correlation coefficient with the formula given below:


(7) $$R\,\left(Y,\hat{Y}\right)=\frac{\sum_{i=1}\left({\hat{Y}}_{i}-\,\bar{Y}\right)\,\,\left(Y_{i}-\,\bar{Y}\,\right)}{\sqrt{\sum_{i=1}{\left({\hat{Y}}_{i}-\,\bar{Y}\right)}^{2}}\,\sqrt{\sum_{i=1}{\left(Y_{i}-\,\bar{Y}\right)}^{2}}}$$

With $\hat{Y}$ defining the predicted values, *Y* being the real values, N is the number of observations and $\,\bar{Y}$ is the average of the real values in *Y*. The RMSE metric measures the standard deviation of the residuals between the predicted and actual targets, while the correlation coefficient metric measures the weight of similarity between them. Low RMSE and high correlation coefficient are desirable, conveying how well the predictive model is approximating the targets.

#### Task 2: Classification Task for Prediction of Disease Status

For the classification task, the subjects were grouped according to the diagnosis label defined by ADNI as AD, EMCI, LMCI, and CN. The diagnosis label has also been tracked and labeled 24th months after their first visit and subjects are then labeled as MCI converter group (MCI-C) if they have been diagnosed as MCI at baseline and their diagnosis status has progressed into AD. The MCI Non-Converter group (MCI-NC) label is assigned to subjects whose diagnosis label did not change after 24 months. The network is trained to perform a 4-way classification (along with the longitudinal regression task) to predict the subjects’ class labels after 24 months. In this second test using the features at baseline, the aim was to predict the probability of converting from MCI to AD, 24 months ahead of time.

## Results

### Prediction Results

**FIGURE 4:**
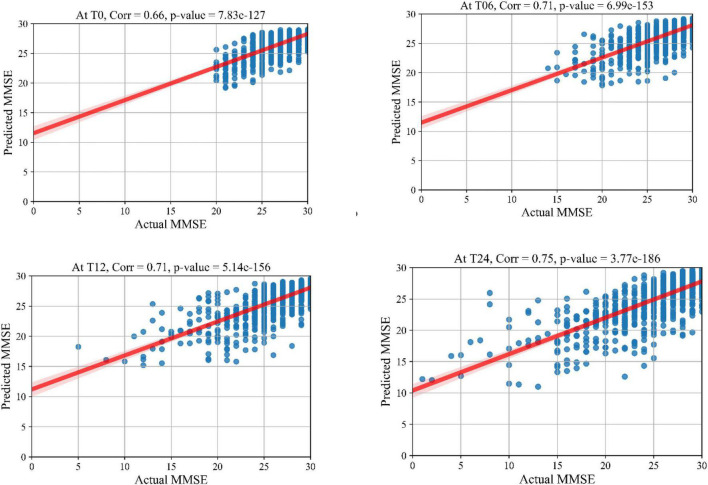
Scatter plots of predicted MMSE values.

The prediction results for the MMSE test scores at baseline and at time points of 6 months, 12 months, and 24 months are summarized in Table 3. In this table, SVR is the conventional Support Vector Regression model. Since models reported in the literature and referenced in this table were using different numbers of features, preprocessing methods, and data modalities, we have taken a similar approach as Abrol et al. (2021) and compared our results with baseline models of SVR, Elastic Net, and Random Forest that were trained and tested using the same data that used to train and test the KTMnet model. The proposed model demonstrated an average RMSE of 2.32 ± 0.52 and a correlation of 0.71 ± 5.98 for predicting MMSE throughout the 24 months after baseline. Figure 4 shows the scatter plots of predicted MMSE values vs. the actual target values at time points T0, T6, T12, and T24.

**TABLE 3:** Comparison of longitudinal regression performance of the proposed network in contrast to other methods reported in the literature.

			T0		T06		T12		T24	
Study	Data	Subjects	RMSE	Corr	RMSE	Corr	RMSE	Corr	RMSE	Corr
Zhu et al. (2016a)	MRI + PET^a^	202	1.80 ± 0.13	0.57 ± 0.23	−	−	−	−	−	−
Liu et al. (2018c)	MRI + DEM^a^	1,984	2.37	0.57	−	−	−	−	−	−
Cao et al. (2018)	MRI^b^	755	2.37 ± 0.19	0.57 ± 0.05	−	−	−	−	−	−
Lei et al. (2017)	MRI^b^	445	1.75 ± 0.20	0.75 ± 0.08	2.31 ± 0.29	0.79 ± 0.10	2.48 ± 0.40	0.79 ± 0.12	3.00 ± 0.38	0.83 ± 0.06
Zhang and Shen (2013)	MRI + PET + CSF^b^	186	2.11 ± 0.35	0.65 ± 0.27	−	−	−	−	−	−
Elastic net	Multimodal*^b^	1,117	1.84 ± 0.35	0.71	2.58 ± 0.34	0.54	2.91 ± 0.53	0.51	3.64 ± 0.56	0.50
SVR	Multimodal*^b^	1,117	1.75 ± 0.44	0.42	2.02 ± 0.53	0.54	2.52 ± 31	0.54	3.12 ± 0.41	0.51
Random forest	Multimodal*^b^	1,117	1.74 ±	0.78	1.98 ± 0.45	0.67	2.36 ± 0.36	0.73	3.15 ± 0.28	0.70
Tabarestani et al. (2020)	Multimodal*^b^	1,620	1.62 ± 0.24	0.82	1.78 ± 0.22	0.86	2.24 ± 0.24	0.80	2.38 ± 0.21	0.81
KTMnet	Multimodal*^b^	1,117	1.79 ± 0.12	0.66 ± 0.81	2.10 ± 0.15	0.71 ± 0.92	2.42 ± 0.28	0.71 ± 0.41	2.97 ± 0.45	0.75 ± 3.10

*Multimodal here refers to using MRI, PET, DEM, CSF, and cognitive measurements without the inclusion of ADAS11, ADAS13, and CDRSB.

*^a^Imaging data.*

*^b^Tabular data.*

### Multiclass Classification Results

In this experiment, the results of the multiclass classification considering the four groups of AD, MCI-C, MCI-NC, and CN are shown in Table 4 with a comparison to other competing methods in the literature. In this multiclass classification process, it is important to investigate the classification performance of the network for each category of subjects. The total classification accuracy achieved by our proposed KTMnet method is 66.85 ± 3.77. In classifying the AD group from all other classes, the proposed network achieved a precision of 70.49% ± 9.33, a sensitivity of 57.21 ± 9.41, an F1 score of 62.72 ± 10.11, and an AUC of 94%. In classifying the MCI-C group, the network reached a precision of 45.33 ± 7.22, a sensitivity of 50.79 ± 9.42, an F1 score of 47.72 ± 7.62, and an AUC of 83%. In classifying the MCI-NC group, the network reached a precision of 69.72 ± 8.63, a sensitivity of 67.57 ± 7.00, an F1 score of 68.16 ± 5.06, and an AUC of 84%. In classifying the CN group, the network reached a precision of 77.89 ± 6.62, a sensitivity of 79.78 ± 9.74, an F1 score of 78.10 ± 5.89, and an AUC of 94%.

**TABLE 4:** Comparison of 4-way multiclass classification performance of methodologies reported in the literature using ADNI dataset.

Study	Data	Subjects	Validation method	Accuracy
Liu et al. (2015) ^a^	MRI	758	10-fold	46.30 ± 4.24
Liu et al. (2015) ^a^	MRI + PET	331	10-fold	53.79 ± 4.76
Zhu et al. (2016a) ^a^	MRI + PET	202	10-fold	0.619 ± 1.54
Liu et al. (2018c)	MRI + PET + DEM^a^	202	Independent test	51.80
Zhu et al. (2016b)	MRI + PET	202	10-fold	61.06 ± 1.40
Zhang and Shen (2013)	MRI + PET + CSF	805	10-fold	53.72 (max)
SVM	MRI + PET + CSF + COG + DEM	1,117	10-fold	58.49 ± 4.01
Random forest	MRI + PET + CSF + COG + DEM	1,117	10-fold	60.28 ± 2.83
KTMnet	MRI + PET + CSF + COG + DEM	1,117	10-fold	66.85 ± 3.77

*^a^DEM stands for Demographic information (Age, Gender, and Education).*

**FIGURE 5:**
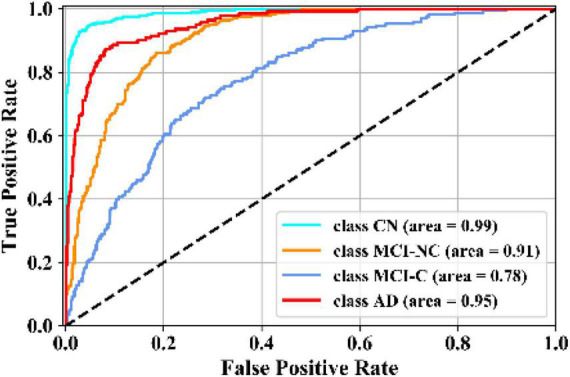
Comparison of ROC curves of the KTMnet for AD vs. MCI-C vs. MCI-NC vs. CN.

**FIGURE 6:**
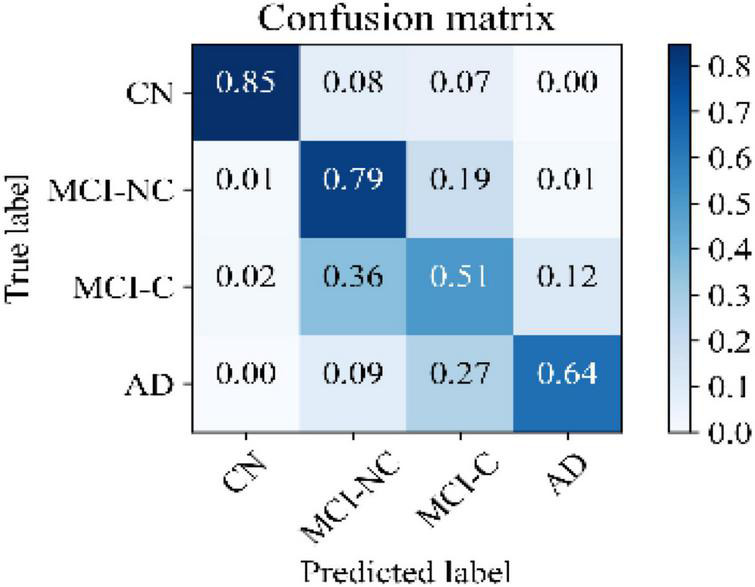
Confusion matrix of the KTMnet model.

Figure 5 illustrates the receiver operating characteristic (ROC) curves showing the capability of the network in discriminating between the four groups. This graph outlines the classification performance over all sets of possible thresholds. By varying the threshold, the observations are assigned to certain classes and the True Positive Rate on the *y*-axis is plotted against the False Positive Rate in the *x*-axis. Figure 6 shows the confusion matrix for contrasting the correct and incorrect predictions. The CN population was the easiest population for the model to deal with, showing the lowest amount of false-positive in the MCI-NC and MCI-NC groups, and absolutely no miss-classification in the AD group. In contrast, the MCI-C represented the most challenging one, where the model confused several samples with the MCI-NC and AD groups. This raised the number of false-positive and false negatives in both the MCI-NC and AD groups and consequently degraded the precision and sensitivity in these two groups. There is currently no clear reason why some patients will stabilize in the MCI stage and others will transition into the AD stage.

### Design Exploration

Three ablation experiments are conducted to evaluate the effectiveness of tensorization. In the first experiment, the tensorization and feature extraction modules (layers *L*_*4*_ through *L*_*9*_) have been removed and data were directly passed from layer *L*_3_to layer *L*_*10*_. In the second experiment, the tensorization modules (layers *L*_*4*_ through *L*_*9*_) have been replaced by a dense layer which transforms the data from layer *L*_*3*_ to a dimension of 100 × 1, and the output of this dense layer is passed onto the layer *L*_*10*_. The third and last experiment is to keep the layer *L*_*4*_ and to pass the results of this layer to layer *L*_*10*_. This configuration keeps the Gaussian kernel dense layer but removes the next tensorization layers. Table 5 summarizes the experimental results for the tasks of classification and regression. For each experiment, training is stopped by monitoring the loss value of the validation set with 30 patience epochs. Considering the results obtained in this study, the proposed KTMnet obtained the best results among different variations of the network structure. *T*-test has been performed between the prediction results of the proposed model and different model structures discussed in this subsection to measure the statistical significance of the results and the resulting *p*-values which were all less than 0.05. The most competing network (in terms of metrics) was the second configuration, where the network was taking advantage of a simple fully connected layer with the dimension of 100 × 1. This means that the Gaussian layer (in the third experiment) without tensorization and feature extraction modules (*L*_*5*_ to *L*_*9*_) becomes less useful. Need to mention that similar experiments to the second experiment have been conducted to explore the effectiveness of adding various hidden layers with different neuron sizes. In terms of RMSE and correlation coefficients metrics, all other configurations have resulted in almost the same performance. *P*-values between these sets of configurations were greater than 0.05 (showing no significant improvements between these results). Therefore, to keep the manuscript concise and easier to follow, only the results of adding a dense layer of size 100 × 1 have been reported in Table 5. Another interesting observation is that KTMnet converged faster and stopped with a smaller number of epochs.

**TABLE 5:** Comparison of different configurations of the proposed model discussed as design exploration study.

	T0		T06		T12		T24		Acc
Experiment	RMSE	Corr	RMSE	Corr	RMSE	Corr	RMSE	Corr	
Design exploration 1	5.93 ± 1.29	0.52 ± 0.32	6.02 ± 1.17	0.50 ± 0.43	5.855 ± 1.30	0.51 ± 0.21	6.45 ± 1.08	0.52 ± 041	60.98 ± 3.07
Design exploration 2	1.84 ± 0.15	0.62 ± 0.27	2.46 ± 0.22	0.61 ± 0.18	2.50 ± 0.25	0.58 ± 0.25	3.17 ± 0.32	0.69 ± 0.38	64.42 ± 4.37
Design exploration 3	2.19 ± 0.20	0.56 ± 0.76	2.39 ± 0.35	0.62 ± 0.31	2.63 ± 0.29	0.62 ± 0.43	3.25 ± 0.32	0.70 ± 035	63.16 ± 5.13
KTMnet	1.79 ± 0.12	0.66 ± 0.81	2.10 ± 0.15	0.71 ± 0.92	2.42 ± 0.28	0.71 ± 0.41	2.97 ± 0.45	0.75 ± 0.31	66.85 ± 3.77

## Discussion

The deep learning network developed in this study, together with its unique architecture, is designed to perform both tasks of multiclass classification and regression simultaneously, predicts disease progression by tracking the MMSE test scores at four consecutive future time points in a time window spanning 24 months and assessing their categorical labels as (AD, MCI-C, MCI-NC, and CN). This objective has been accomplished through extracting and fusing the complex inter- and intra-modality features, extracting hidden features by using tensorization that projects the feature space into a higher-dimensional space, and eventually modeling the feature representation through non-linear transformations.

In the reported literature, binary classification of AD patients (including the converter and non-converter groups) has been taken into consideration (Moradi et al., 2015; Hojjati et al., 2017; Liu et al., 2017; Spasov et al., 2019). In these studies, attention was more focused on correctly classified subjects by measuring and reporting the metrics of sensitivity and specificity. However, the more challenging multiclass classification of AD cohorts using multimodal screening tests has not been fully explored for the diagnosis and prognosis of AD. This topic becomes even more challenging when progression is assessed in a population of subjects without any preliminary information about their baseline disease category. In a multiclass classification scenario, where there is no auxiliary information to reduce the number of false-positive and false-negative samples, the probability of over and under diagnosis will be increased, making it more important to use additional metrics for performance evaluation purposes. Table 6 summarizes specific studies that performed multiclass classification or longitudinal regression tasks for meaningful comparisons.

**TABLE 6:** Summary of prediction tasks accomplished in the literature.

Method	Multitask	Classification type	Class name	Regression type	Modality	Subjects
Natarajan et al. (2014)	No	Multiclass	AD-MCI-CN	−	MRI	397
RELM (Natarajan et al., 2014)	No	Multiclass	AD-MCI-CN	−	MRI	214
Zhu et al. (2016b)	No	Multiclass	AD/MCI/CN and AD/MCI-C/MCI-NC/CN)	−	MRI—PET	202
JRMI (Zhu et al., 2016a)	Yes	Multiclass	AD/MCI/CN and AD/MCI-C/MCI-NC/CN	Single time point	MRI—PET	202
DM2L (Liu et al., 2018c)	Yes	Binary and multiclass	AD/MCI/CN and AD/pMCI/sMCI/CN	Single time point	MRI—Demographic	1,984
DW-S2MTL (Suk et al., 2016)	No	Binary and multiclass	AD/MCI/CN and AD/pMCI/sMCI/CN	−	MRI—PET—CSF	805
SMKMTL (Cao et al., 2018)	No	Binary	AD/MCI-C/MCI-NC/CN	Multiple cognitive scores	MRI	788
SAE (Liu et al., 2015)	No	Multiclass	AD/MCI-C/MCI-NC/CN	−	MRI and (MRI + PET)	758–331
SMTL (Lei et al., 2017)	No	−	AD/MCI/CN	4 time points	MRI	445
MSMT (Nie et al., 2017)	No	−	CN/MCI/AD	4 time points	Multimodal	818
CNN (Liu et al., 2018a)	No	Binary	AD/pMCI/sMCI/CN	−	MRI + PET	397
M3T (Zhang and Shen, 2013)	Yes	Binary	MCI-C/MCI-NC and AD/CN and MCI/CN	2y changes of MMSE	MRI + PET + CSF	186
MSJL (Zhu et al., 2014)	No	Binary	AD/CN, MCI/CN, MCI-C/MCI-NC	Single time point	MRI + PET + CSF	202

A noteworthy observation made on this model was the see-saw effect encountered during hyperparameter searching. Although we received better results in comparison to other methods reported in the literature, the classification and regression tasks were not in sync with each other. To be more specific, the regression task was falling from its optimum point when the parameters were tuned to increase classification accuracy, and the reverse was also true when the parameters were tuned for increasing prediction accuracy. This new study suggests that when adjusting the hyperparameters to maximize the results of a first given task (e.g., classification), may not necessarily yield a maximized accuracy in the second task (e.g., prediction), proving a breakpoint from which the same set of hyperparameters is to optimize both prediction and multiclass classification in a multitask framework. Other important issues that complicate this multitask process relate to (1) the imbalance in the number of subjects in each of the subgroups considered (NC, MCI-C, MCI-NC, AD), (2) the fact that the process involves multiclass classification involving the aforementioned 4 subgroups, and the prediction that is performed at all-time points of the longitudinal study.

Another set of experiments was conducted to test the full potential of the proposed network (this time as a single task model). By removing one of the two dense layers in L10, the network had its full degree of freedom to optimize its parameters for only one of the regression or classification tasks. When trained for regression task only, the results show an RMSE of 1.74 ± 0.13, 2.09 ± 0.14, 2.46 ± 0.19, and 3.10 ± 0.26 for T0, T6, T12, and T24, respectively, with an average mean RMSE score of 2.35 ± 0.53 for all four time points. The results are close to the results obtained from the network when it was trained and tested in a multitasking mode. To analyze the significance of the difference between the regression results of the single task and multitask model, the *p-*value between the predicted MMSE scores of the regression-only model with its counterpart predicted MMSE scores from the KTMnet model has been calculated. The *p*-values for all time points were bigger than 0.05, showing no significant difference when the model is optimized to only perform the regression task.

Similarly, another set of experiments has been repeated, aimed at the task of classification. This time, when the model has been set up as a single-task classification model, the model achieved an accuracy of 65.53 ± 3.75 which is again close to the multitask KTMnet accuracy, which is 66.85 ± 3.77. This demonstrates that, although the KTMnet model shows a see-saw effect when being optimized as a multitask model, seemingly unable to reach an ideal point in which both regression and classification tasks are each optimized to their full potential performance, the multitask learning approach is indeed helpful. The first supporting reason for this last assertion is that when the model is designed to only do a single task of regression or classification, it is not able to pass the local optima and achieve better results than with a multitask model. Another supporting factor is the fact that to get classification and regression results from a single task model, two separate models need to be trained, which almost doubles the number of computations that are needed to perform both tasks separately.

The initial expectation from this experiment was that diagnostic labels and cognitive tests should be able to substitute for one another, i.e., they should be able to transform the feature space when being used as targets for a specific model. However, this was not the case in this study. While setting up the experiments, we also tested for the applicability of the model to predict other cognitive tests. Among all these three cognitive scores of (MMSE, RAVLT, and ADAS11) the best results were obtained with multitasking MMSE with diagnosis labels. Thus, we focused on reporting the results of this setup only.

**FIGURE 7:**
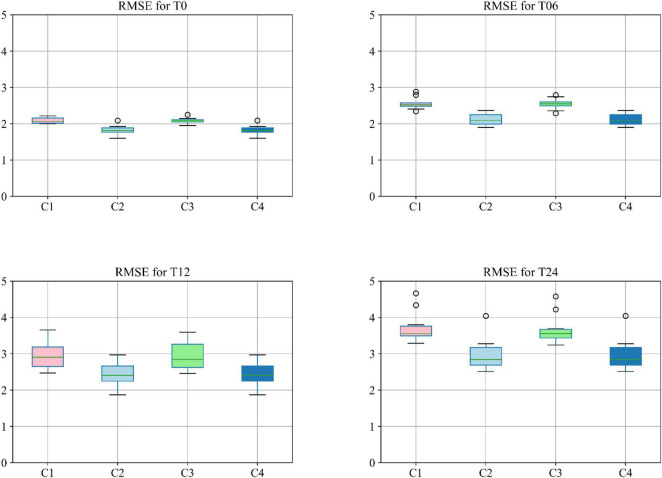
Boxplot for RMSE of mixture category of subjects using different combinations of modalities. Here C1 stands for MRI + PET + RF, C2 stands for MRI + PET + RF + COG, C3 stands for MRI + PET + RF + CSF, C4 stands for MRI + PET + RF + COG + CSF.

Moreover, different combinations of modalities have been investigated to provide for more meaningful comparisons with other reported studies. Results provided in Figure 7 demonstrate the influence of the different combinations of modalities in predicting the MMSE scores. Four different modality combinations have been considered, where RF signifies risk factor, with C1–C4 referring to the various combinations of the different modalities as indicated in the legend of Figure 7.

**FIGURE 8:**
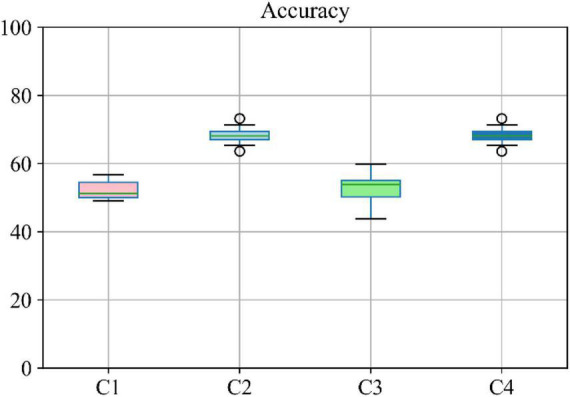
Boxplot for accuracy of multiclass classification achieved through the proposed network based on a different combination of modalities. Here C1 stands for MRI + PET + RF, C2 stands for MRI + PET + RF + COG, C3 stands for MRI + PET + RF + CSF, C4 stands for MRI + PET.

Moreover, the accuracy of the multiclass classification for predicting the progression of AD in a period of 24 months in terms of their categorical labels is shown in Figure 8. It should be noted that for the sake of uniformity, all the results reported in this study are generated using the same network shown in Figure 3. Therefore, the network that has been analyzed to yield the results shown in Figures 7, 8 used the hyperparameters (optimizer, dropout rate, decay rate, hidden layer size, and so on) that have been optimized exclusively with respect to the five modalities considered (MRI, PET, CSF, COG, and DEM).

## Conclusion

In this study, a novel neural network structure with multitask learning, modality fusion, kernelization, and tensorization has been proposed to predict and classify the different stages of Alzheimer’s disease in a multiclass population. Using the features collected at baseline, this newly developed network is shown to predict the cognitive status (through the MMSE scores) of the patients in a 24-month longitudinal study involving the AD/MCI-C/MCI-NC/CN groups [taking into consideration the converter (C) and non-converter groups (NC) in the MCI category]. Multitask learning has been explored to enhance prediction performance by incorporating the common relationship or interrelatedness between the regression and multiclass classification tasks. Furthermore, the power of modality fusion, kernelization, and tensorization have also been investigated to efficiently extract important features hidden in the lower-dimensional feature space without being distracted by those deemed irrelevant.

Empirical evaluations on the longitudinal multimodal ADNI dataset were conducted in this study to evaluate the model’s performance. The results reveal that the proposed KTMnet framework not only predicts the cognitive scores with relatively high accuracy but can also enhance the multiclass classification accuracy for early stage diagnosis and prognosis of the MCI conversion group. It is emphasized here that although we are aware of the overlap that exists in the MMSE scores in between subject groups, making the prediction of MMSE scores difficult, we still removed from consideration in the training phase the predictive biomarkers of ADAS13, MoCA, and CDR, which are found to be highly correlated to MMSE. Their inclusion otherwise would have favored the proposed machine learning design and could have biased the accuracy for both prediction and multiclass classification.

In relation to Figure 2, for each cognitive test (RAVLT, MMSE, ADAS11) and each subgroup, this study shows that there may be a learning effect at 12 months, which continues to 24 months for CN and MCI-NC; however, for the AD and MCI-C groups, the learning effect seems to be overtaken by the disease effect beyond year 1.

## Data Availability Statement

The original contributions presented in the study are included in the article/supplementary material, further inquiries can be directed to the corresponding author/s.

## Author Contributions

ST and ME designed and developed the model. ST analyzed the data, performed the statistical analysis, and interpreted the data. MA provided overall guidance on the design of the model. ST, ME, and MA wrote the manuscript. RD, SD, and DV provided clinical input on the multimodal framework of the model. RD provided input on the structure of the article. DL and RC provided input on the merits of the cognitive tests. AB, MC, and NR provided input on methodology and problem description. All authors revised the manuscript for important scientific content and final approval.

## Conflict of Interest

The authors declare that the research was conducted in the absence of any commercial or financial relationships that could be construed as a potential conflict of interest.

## Publisher’s Note

All claims expressed in this article are solely those of the authors and do not necessarily represent those of their affiliated organizations, or those of the publisher, the editors and the reviewers. Any product that may be evaluated in this article, or claim that may be made by its manufacturer, is not guaranteed or endorsed by the publisher.
